# Fast detection of moisture content and freshness for loquats using optical fiber spectroscopy

**DOI:** 10.1002/fsn3.4130

**Published:** 2024-04-14

**Authors:** Qinglong Meng, Shunan Feng, Tao Tan, Qingchun Wen, Jing Shang

**Affiliations:** ^1^ School of Food Science and Engineering Guiyang University Guiyang China; ^2^ Research Center of Nondestructive Testing for Agricultural Products of Guizhou Province Guiyang China

**Keywords:** freshness, loquats, moisture content, non‐destructive detection, optical fiber spectroscopy

## Abstract

Detection of the moisture content (MC) and freshness for loquats is crucial for achieving optimal taste and economic efficiency. Traditional methods for evaluating the MC and freshness of loquats have disadvantages such as destructive sampling and time‐consuming. To investigate the feasibility of rapid and non‐destructive detection of the MC and freshness for loquats, optical fiber spectroscopy in the range of 200–1000 nm was used in this study. The full spectra were pre‐processed using standard normal variate method, and then, the effective wavelengths were selected using competitive adaptive weighting sampling (CARS) and random frog algorithms. Based on the selected effective wavelengths, prediction models for MC were developed using partial least squares regression (PLSR), multiple linear regression, extreme learning machine, and back‐propagation neural network. Furthermore, freshness level discrimination models were established using simplified k nearest neighbor, support vector machine (SVM), and partial least squares discriminant analysis. Regarding the prediction models, the CARS‐PLSR model performed relatively better than the other models for predicting the MC, with *R* ^2^_P_ and RPD values of 0.84 and 2.51, respectively. Additionally, the CARS‐SVM model obtained superior discrimination performance, with 100% accuracy for both calibration and prediction sets. The results demonstrated that optical fiber spectroscopy technology is an effective tool to fast detect the MC and freshness for loquats.

## Graphical abstract

The developed CARS‐PLSR prediction model performed relatively better than the other models for predicting the moisture content of loquats, with RPD values of 2.51. The CARS‐SVM discrimination model for determining the freshness of loquats obtained superior discrimination performance, with 100% accuracy for both calibration and prediction sets. This study demonstrated that optical fiber spectroscopy technology is an effective tool to fast detect the moisture content and freshness for loquats.

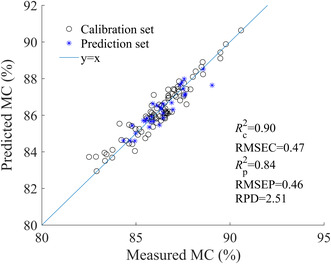

## 1 INTRODUCTION

The loquat (*Eriobotrya japonica* [Thunb.] Lindl.) is a subtropical evergreen fruit tree belonging to the family Rosaceae, specifically the subfamily Pomoideae, native to southeast China (Undurraga et al., 2011). It is characterized by a rich source of phenolics and carotenoids (Li, Xu, & Chen, 2016). Furthermore, cough and reducing phlegm which is particularly significant, so it is deeply loved for its dual‐purpose medicine and food (Huang et al., 2021). Currently, there is a rising demand for fresh fruit attributed to an increasing awareness of the importance of nutrition and the adoption of healthier lifestyles (Armghan Khalid et al., 2022; Li & Thomas, 2014). Fresh loquats are prone to water loss, leading to wilting, shriveling, and browning, which reduces their shelf‐life and causes significant economic loss (Lufu et al., 2020; Pareek et al., 2014; Shah et al., 2023; Tian et al., 2011). Therefore, assessing the moisture content (MC) of loquats is a crucial step in ensuring their freshness and commercial value.

Detection of the MC by traditional instrumental techniques is often destructive. Consequently, the development of non‐destructive techniques is essential for determining the MC and freshness level of loquats (Li, Huang, et al., 2022; Shang et al., 2023). Non‐destructive detection methods that combine spectroscopic techniques with chemometrics offer several advantages, including fast analysis, no environmental pollution, and no damage (Li, Zhang, & Wang, 2022). In recent years, this method has gained widespread use in assessing the quality of fresh fruit (Ye et al., 2022; Yildiz et al., 2022). At present, researchers have conducted studies on quality detection in various fruits, including kiwifruit (Xu et al., 2023), apple (Pissard et al., 2021), grape (Kanchanomai et al., 2020), watermelon (Ibrahim et al., 2022), strawberry (Zhao et al., 2023), and others. Minas et al. (2021) conducted accurate assessment of peach internal quality and physiological maturity using near infrared spectroscopy (NIRS), which is helpful to facilitate the wider application of NIRS throughout the tree fruit supply chain. Sun et al. (2020) developed a non‐destructive detection method for determining blackheart pear and soluble solids content. The partial least square discriminate analysis model achieved a discrimination rate of 96.88% for identifying blackheart pears, while the partial least squares model calibrated with healthy pears exhibited improved performance with a root mean square error in prediction set of 0.45. Alhamdan and Atia (2017) investigated the application of near infrared spectroscopy for assessing the quality of Barhi dates at different maturity stages. The proposed models achieved the coefficient of determination values of 0.97, 0.94, and 0.64 for total soluble solids, MC, and b* color, respectively. Tantinantrakun et al. (2023) demonstrated that both transmittance short wavelength near infrared spectroscopy (SW‐NIRS) and reflectance near infrared hyperspectral imaging (NIR‐HSI) could determine the maturity index in pineapples. The SW‐NIRS achieved a coefficient of determination considering cross‐validation (*R* ^2^_cv_) of 0.70, while the NIR‐HSI showed excellent prediction with an *R* ^2^_cv_ of 0.72. Specifically, optical fiber spectroscopy, with its low cost and rapid measurement, has become a widely used technology for assessing fruit quality (Li, Sun, & Cheng, 2016). Tewari et al. (2008) combined optical fiber spectroscopy with machine learning algorithms to determine the origin and sugars of citrus fruits. Kawano et al. (1992) investigated the feasibility of fiber optics in interactance mode to predict sugar content in intact peaches. Guthrie and Walsh (1997) proposed modified partial least squares (MPLS) regression for analyzing pineapple juice Brix and mango flesh dry matter by near infrared spectroscopy. The MPLS models achieved a multiple coefficient of determination of 0.91 for pineapple juice Brix and 0.98 for mango flesh dry matter. These studies indicated that the feasibility of determining fruit quality using optical fiber spectroscopy techniques. However, there are no studies reporting the simultaneous determination of the MC and freshness for loquats using optical fiber spectroscopy technology.

In this study, optical fiber spectroscopy techniques combined with chemometrics was applied to explore the feasibility of determining the MC and freshness for loquats. The main objectives were (1) to establish prediction models for determining the MC of loquats including partial least squares regression (PLSR), multiple linear regression (MLR), extreme learning machine (ELM), and back‐propagation (BP) neural network, (2) to develop discrimination models for different freshness levels of loquats including simplified k nearest neighbor (SKNN), support vector machine (SVM), and partial least square discrimination analysis (PLS‐DA), (3) to select feature variables from full spectra using competitive adaptive reweighted sampling (CARS) and random frog (RF) methods for designing simplified models.

## 2 MATERIALS AND METHODS

### 2.1 Sample preparation

**FIGURE 1:**
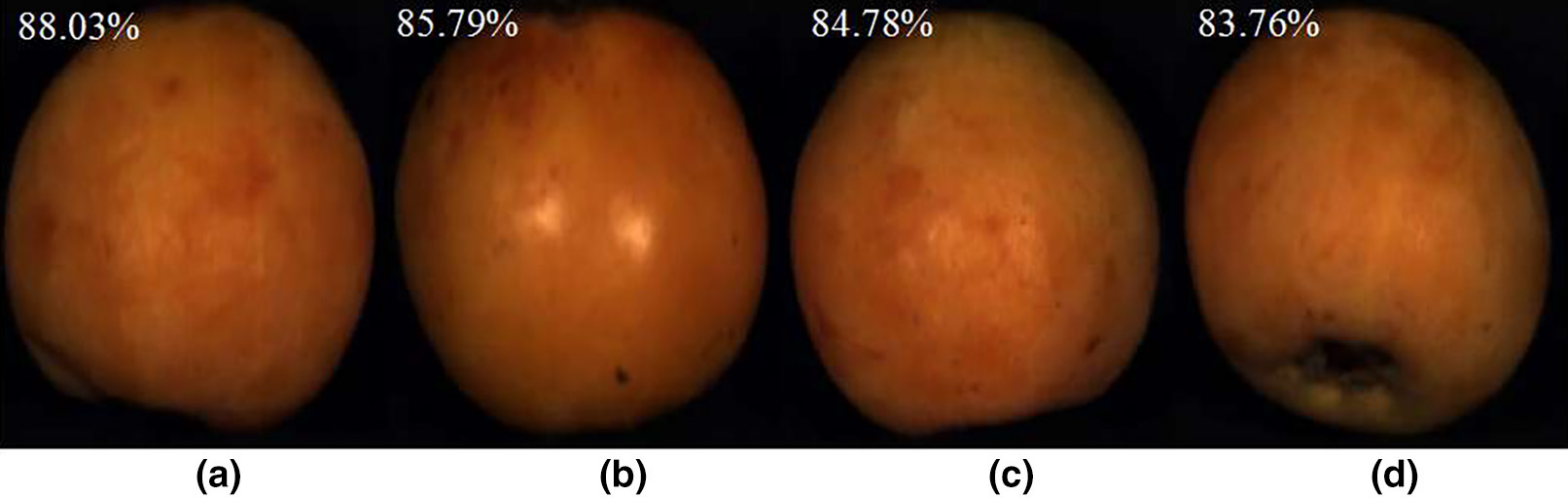
Images of the loquat samples stored for 1 (a), 2 (b), 3 (c) and 4 (d) days.

The fresh loquat samples were harvested from the commercial orchards (Loquat Green Planting Demonstration Garden of Kaiyang County) located in Guizhou Province, China, on June 7, 2022. A total of 120 loquats were collected for the experiment after removing defective loquats with bruises and deformities. The collected samples were then numbered and stored in a laboratory environment at 23 ± 2°C. The experiment commenced 1 day later and continued for 4 days. Each day, 30 samples were used for the experiment. During the experiment, the spectra of each sample was acquired, and their MC was subsequently measured. The images of the loquat samples at the four different storage time were shown in Figure 1.

### 2.2 Spectra acquisition

**FIGURE 2:**
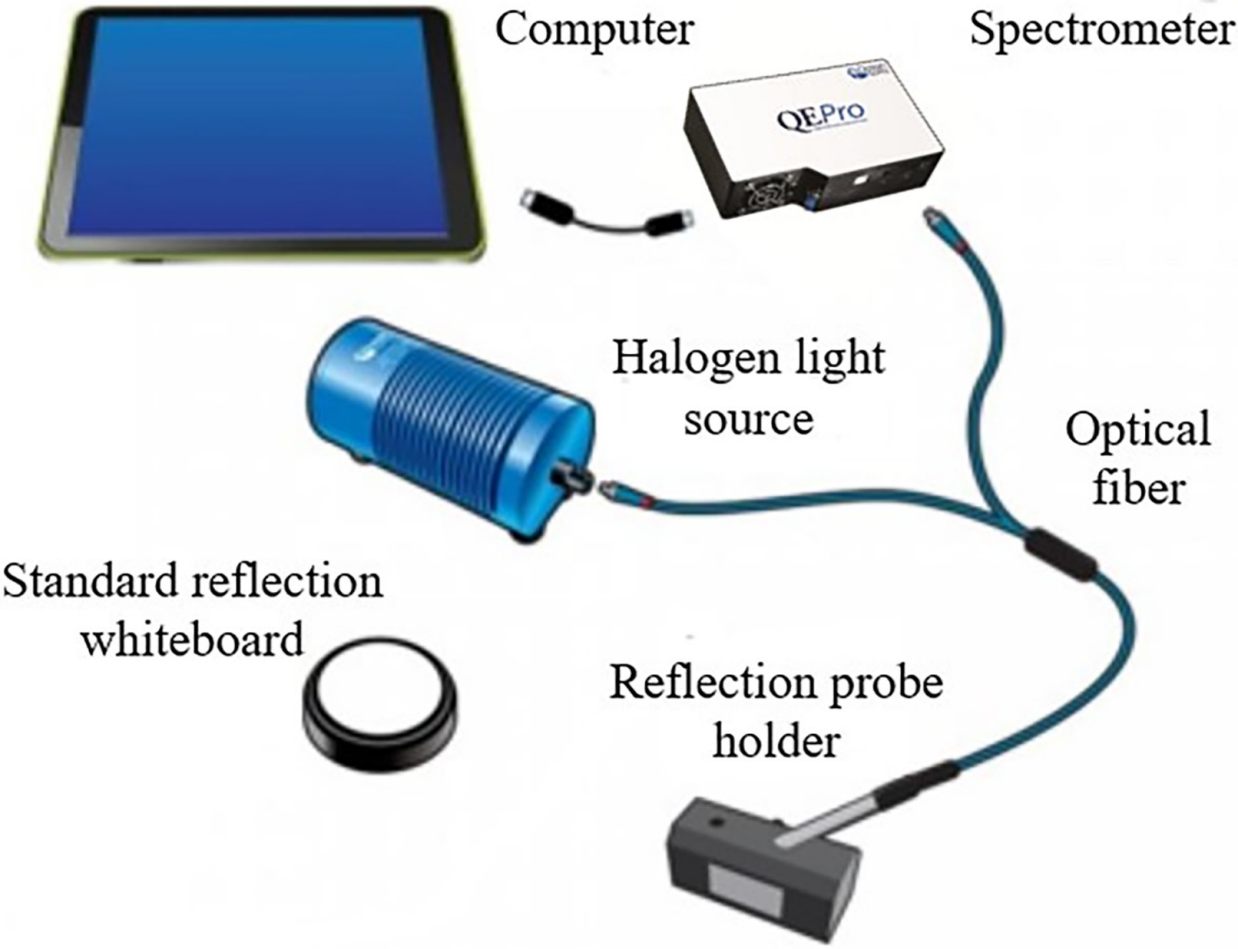
Schematic diagram of optical fiber spectroscopy acquisition system.

The schematic diagram of optical fiber spectroscopy acquisition system (Weihai Optical Instrument (Shanghai) Co., Ltd.) was shown in Figure 2. It mainly included a spectrometer (QE*Pro*, wavelength range: 200–1000 nm, spectral resolution: 2.84–3 nm), optical fiber (R600‐7‐VIS‐125F, diameter: 600 μm), halogen light source (HL‐2000, wavelength range: 360–2400 nm), reflection probe holder (RPH‐1), adapter (RPH‐ADP), standard reflection whiteboard (WS‐1), and a special computer equipped with spectral data acquisition software of Ocean View.

The light source was preheated for 30 minutes prior to spectrometry. Experiments were conducted to adjust parameters, including integration time and averaging times, based on the spectral intensity of the test standard reflectance whiteboard. The parameters of the system were: the integration time of 110 ms, the average number of scans of 8, and the average sliding width of 1. The reflection probe was attached to the RPH‐1 using the RPH‐ADP. The surface of RPH‐1 was positioned 1 cm away from the loquat sample. Subsequently, the tested loquat sample was tightly placed on the surface of RPH‐1, and spectra were collected from equatorial positions in each sample.

### 2.3 Moisture content measurement

The loquats were sliced meticulously into thin slices with an approximate thickness of 3 mm using a sharp knife. The slices were evenly distributed in a weighing dish, and the initial weight of each sample was continuously recorded using a highly accurate balance (accuracy ±0.001 g). Subsequently, the weighing dish was then placed in a drying oven (101–2, Tianjin Taisite Instrument Co., Ltd., China) and heated at a temperature of 75°C for 8 h. The dish was then cooled to room temperature after the heating period, in preparation for subsequent weighing. Additionally, the fruit slices were heated for an additional 2 hours, cooled, and then weighed. The process was repeated until the absolute difference between two consecutive weighings did not exceed 0.001 g, signifying that the fruit had achieved a state of constant weight. The MC was calculated by the following equation (Crichton et al., 2018):

(1) $$X=\frac{w_{1}-w_{2}}{w_{1}-w_{0}}\times 100\%$$

where, *X* represents the MC of loquats. *w*_0_ denotes the weight of the empty weighing dish in g. *w*_1_ signifies the weight of the weighing dish and the sample before oven drying in g. *w*_2_ indicates the weight of the weighing dish and the sample after oven drying in g.

### 2.4 Spectra pre‐processing algorithm

Spectral data acquired from the spectrometer not only contains valuable sample information but also includes background information and noise. To eliminate interference information and enhance the predictive ability, it is essential to pre‐process spectral data before modeling (Zhang et al., 2022). In this study, the obtained reflectance spectra were pre‐processed using standard normal variate (SNV), a row‐oriented transformation that centers and scales individual spectra (Guo et al., 2016). SNV possesses the capability to mitigate the influence of particle size, surface scattering, and optical path variations on the spectra (Xiao et al., 2022). The spectra are pre‐processed using the following formula:

(2) $$\left\{\begin{array}{l} Z_{\mathrm{ij}}=\frac{x_{\mathrm{ij}}-{\bar{x}}_{i}}{S_{i}}\\ {\bar{x}}_{i}=\frac{1}{p}\sum_{j=1}^{p}x_{\mathrm{ij}}\\ S_{i}=\sqrt{\frac{1}{p-1}\sum_{j=1}^{p}{\left(x_{\mathrm{ij}}-{\bar{x}}_{i}\right)}^{2}} \end{array}\right.$$

where, *Z*_*ij*_ represents spectra after pre‐processing, *x*_*ij*_ is raw spectrum, ^−^ *x*_*i*_ represents average spectrum, *S*_*i*_ represents the standard deviation of the *i* sample spectral data, and *p* is number of spectral points.

### 2.5 Kennard‐Stone algorithm

In this study, samples were divided into a calibration set and a prediction set using the Kennard‐Stone (KS) algorithm. The KS algorithm starts by selecting a pair of samples with the largest Euclidean distance. Sequentially, a sample is chosen, which exhibits the greatest minimum Euclidean distance between the previously selected samples and the remaining samples. This process continues until the desired number of samples is achieved (Wang & Wang, 2022).

### 2.6 Effective variables selection algorithms

Each spectrum within the dataset consist of 1024 variables. Several studies have demonstrated that efficient wavelength selection can simplify calibration modeling and enhance the accuracy and robustness of the model (Li & Chen, 2017). This study employed CARS and RF to select effective variables.

Competitive adaptive weighting sampling algorithm (Tang et al., 2021) is a variable selection method that applies the principle of “survival of the fittest”. It employs the adaptive reweighted sampling technique to address collinear and non‐information variables. The variables are determined based on the absolute value of the PLSR regression coefficient. Individuals with larger regression coefficients are retained, while those with smaller coefficients are eliminated during the selection process. The optimal subset of variables is determined through the cross‐validation method, which aims to minimize the root mean square error of cross‐validation (RMSECV) (Li et al., 2021).

RF algorithm is initially proposed for the analysis of gene expression in diseases. It is similarities with reversible jump Markov chain Monte Carlo, which simulates a Markov chain in the model space to calculate variable weights according to a steady‐state distribution. The greater the importance of a variable to the model, the higher its probability of being selected. As a result, it is possible to rank the selection probabilities of all variables and choose the one with the highest probability as the characteristic wavelength. To mitigate the influence of random factors, multiple runs are necessary, and the results are tallied (Zhang et al., 2019).

### 2.7 Quantitative modeling and evaluation

Four quantitative models, namely PLSR, MLR, ELM, and BP neural network, were utilized to predict the MC of loquats using the selected characteristic spectra.

Partial least squares regression is a highly reliable linear method widely used for establishing predicted quality parameters in food products. It is capable of addressing multiple covariance issues and handling a larger number of variables than the sample size, demonstrating its predictive potential (Shao et al., 2022). MLR is a statistical method used for regression analysis, offering the advantages of simplicity, low computational complexity, and high accuracy in fitting (McCann et al., 2010). It aims to establish a linear regression model by correlating two or more explanatory variables with a response variable (Kamruzzaman et al., 2013). ELM algorithm is designed for training single hidden layer feedforward neural networks (Wang et al., 2022). It exhibits rapid learning and superior generalization performance compared to traditional feedforward neural network algorithms (Li et al., 2019). BP neural network is a multilayer feedforward neural network trained iteratively using the error back‐propagation algorithm. Classical BP neural networks consist of input, hidden, and output layers (Qi et al., 2021). These networks employ forward propagation and backward error propagation to calculate activation values and update the weights of neurons in each layer (Buscema, 1998).

The performance of the calibration model was assessed using the calibration determination coefficient (*R* ^2^_C_) and root mean square errors (RMSEC). To evaluate the prediction ability of the model, the prediction determination coefficient (*R* ^2^_P_), root mean square errors (RMSEP), and residual prediction deviation (RPD) were calculated. Usually, a well‐performing model exhibits higher values of *R* ^2^_C_, *R* ^2^_P_, and RPD, and lower values of RMSEC and RMSEP. A model is considered poor‐performing when the RPD is below 1.5, whereas an RPD ranging from 1.5 to 2 indicates moderate performance. An RPD between 2 and 2.5 suggests good performance, while an RPD exceeding 2.5 indicates excellent performance (Askari et al., 2015).

(3) $$R_{C}^{2}=1-\frac{\sum_{i=1}^{n_{c}}{\left[y_{\mathrm{act}}\left(i\right)-y_{\mathrm{cal}}\left(i\right)\right]}^{2}}{\sum_{i=1}^{n_{c}}{\left[y_{\mathrm{act}}\left(i\right)-\text{ymean}\left(i\right)\right]}^{2}}$$

(4) $$R_{P}^{2}=1-\frac{\sum_{i=1}^{n_{p}}{\left[y_{\mathrm{act}}\left(i\right)-\text{ypre}\left(i\right)\right]}^{2}}{\sum_{i=1}^{n_{p}}{\left[y_{\mathrm{act}}\left(i\right)-\text{ymean}\left(i\right)\right]}^{2}}$$

(5) $$\text{RMSEC}=\sqrt{\frac{1}{n_{C}}\sum_{i=1}^{n_{c}}{\left[y_{\mathrm{act}}\left(i\right)-y_{\mathrm{cal}}\left(i\right)\right]}^{2}}$$

(6) $$\text{RMSEP}=\sqrt{\frac{1}{n_{p}}\sum_{i=1}^{n_{p}}{\left[y_{\mathrm{act}}\left(i\right)-\text{ypre}\left(i\right)\right]}^{2}}$$

(7) $$\mathrm{RPD}=\frac{\mathrm{SD}}{\text{RMSEP}}$$

where, *n*_c_ is the number of samples in the calibration set, *n*_*p*_ is the number of samples in the prediction set. *y*_*act*_ and *y*_*mean*_ are the referenced and average values, respectively. *y*_*cal*_ and *y*_*pre*_ are the predicted values in the calibration and prediction sets, respectively. SD is the standard deviation of the referenced values in the prediction set.

### 2.8 Qualitative modeling and evaluation

Three qualitative models, namely SKNN, SVM, and PLS‐DA, were established to identify freshness levels of loquats using the selected characteristic spectra.

Simplified k nearest neighbor operates by initially computing the center of gravity of each category in the calibration set and then calculates the distance between each sample in the prediction set and the center of gravity of each respective category. The smaller the distance, the more likely it is to fall into the category. SVM is a popular algorithm widely employed in pattern recognition (Ning et al., 2017). Its objective is to build a hyperplane or set of hyperplanes in a high‐dimensional space to achieve optimal separation between different sample classes (Sanz et al., 2016). PLS‐DA combines the properties of PLS regression with a classification technique in order to establish a relationship between the spectral data variables in the X block and the represented classes in the y vector (Bonifazi et al., 2019).

The evaluation of discrimination results used a confusion matrix to evaluate the performance of the discrimination models. The discrimination rate was also used to estimate the performance of the discrimination models, as follows (Teye et al., 2013):

(8) $$I_{R}=\frac{N_{1}}{N_{2}}\times 100\%$$

where, *I*_*R*_ represents the discrimination rate, *N*_1_ represents the number of correctly discriminated samples, and *N*_2_ represents the total number of samples.

## 3 RESULTS AND DISCUSSION

### 3.1 Spectral analysis

**FIGURE 3:**
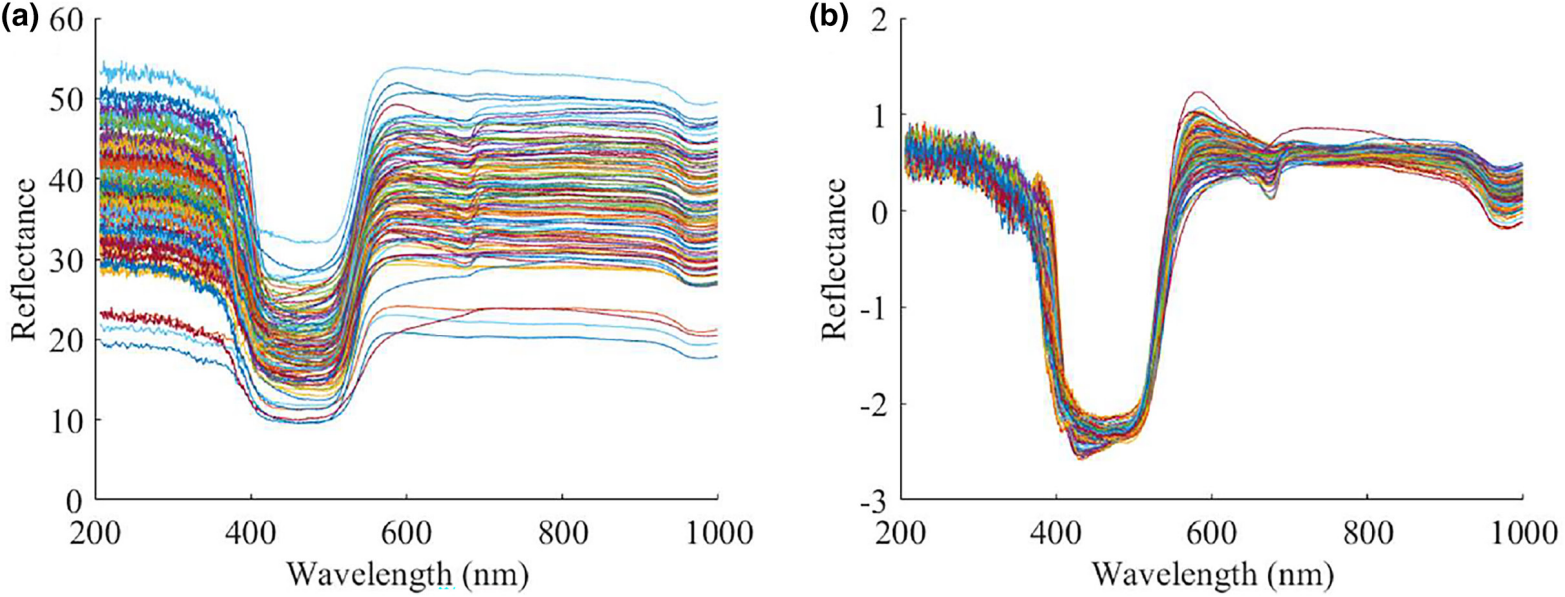
Reflectance curves of raw spectra (a) and SNV pre‐processed spectra (b).

The raw and pre‐processed spectra of all loquat samples in the range of 200–1000 nm were shown in Figure 3. The spectra presented a consistent tendency, but their reflection intensities were different. After SNV pre‐processing, the differences in spectral curves between the loquat samples were reduced and the overall curves became smoother. The absorption peak at 675 nm was primarily attributed to chlorophyll absorption on the surface of the loquat, resulting in the color appearance of loquats (Munera et al., 2021). Furthermore, the absorption peak at 980 nm was primarily attributed to water absorption in the loquat, indicating its water content (Camps & Christen, 2009).

### 3.2 Statistics of moisture content

**FIGURE 4:**
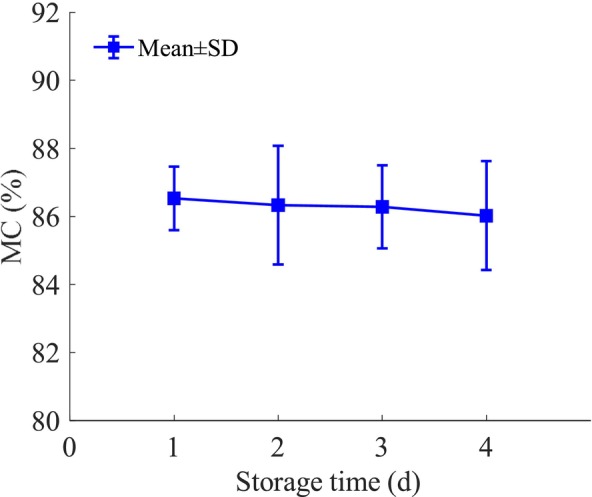
MC of the loquat samples at the different storage time.

The Moisture content of loquat samples at different storage time was shown in Figure 4, represented as the values of mean ± SD (standard deviation). A decreasing trend was observed in the MC of loquat samples with the increasing of storage time.

The KS algorithm was used to divide 120 fresh loquats into the calibration set (*n* = 90) and the prediction set (*n* = 30) with a 3:1 ratio. Table 1 showed the statistics of the MC of all loquat samples. As can be seen, the calibration set exhibited a wider range (82.51–90.59%) for the MC compared to the prediction set. This finding suggests the reasonableness of the results and the high representativeness of the selected modeling samples.

**TABLE 1:** Statistics of the MC of loquats.

Sample set	Number	Min	Max	Mean	SD
Total	120	82.51	90.59	86.29	1.43
Calibration set	90	82.51	90.59	86.27	1.52
Prediction set	30	84.34	89.05	86.35	1.14

### 3.3 Modeling based on feature variables by CARS

**FIGURE 5:**
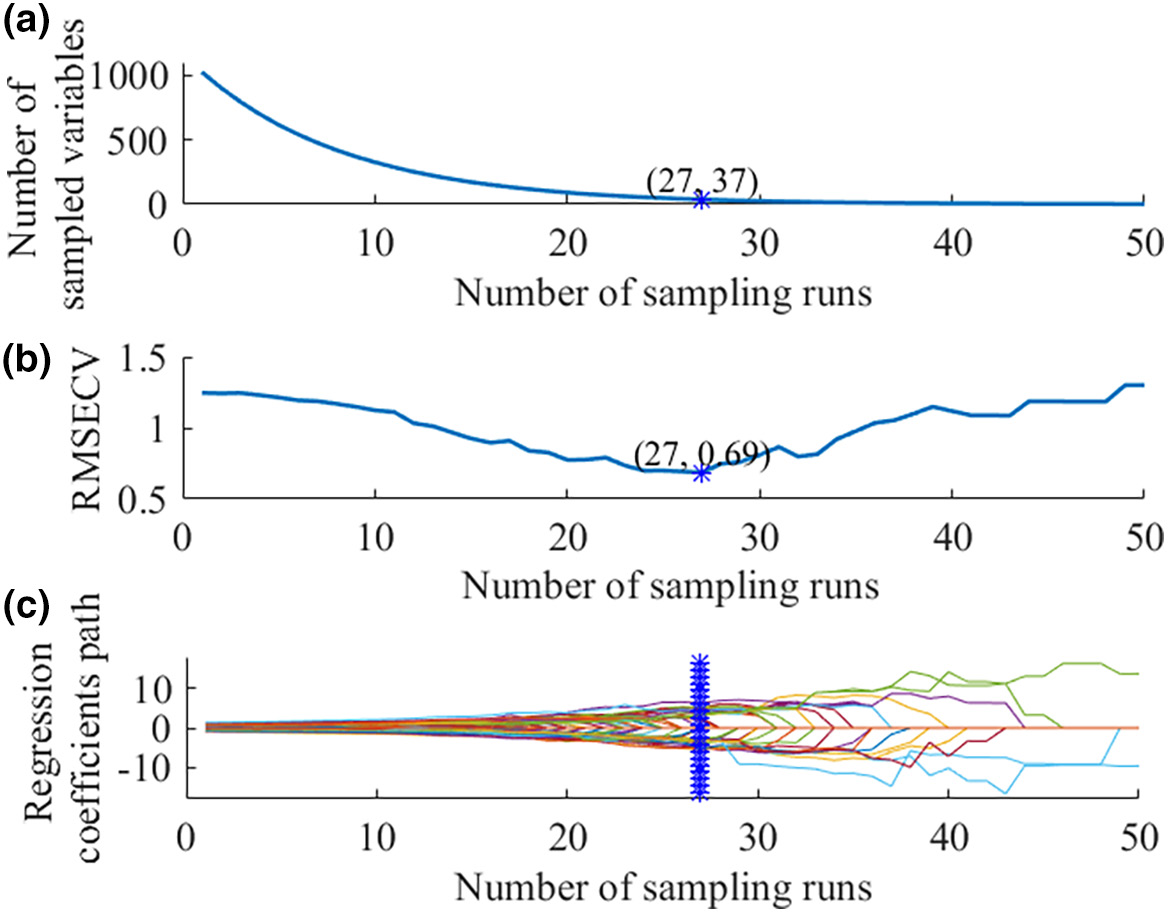
Processing of CARS for the MC. The change trend of the variables number (a), 5‐fold RMSECV values (b), and the regression coefficient path (c) with the increase of Monte‐Carlo sampling runs.

For CARS, 50 Monte Carlo sampling runs were conducted to determine the optimal feature wavelengths by using 5‐fold cross‐validation. Uninformative wavelengths were removed, while the effective wavelengths were remained. Figure 5a–c presented the changes in the number of sampled wavelengths, 5‐fold RMSECV values, and regression coefficients for each wavelength as the number of sampling runs increases.

In Figure 5a, there was a rapid drop followed by a slower decline in the sampling wavelength, indicating the implementation of both rapid and fine selection in CARS. In Figure 5b, the RMSECV values gradually declined from sampling runs 1 to 27 as the number of sampling runs increased, mainly because uninformative wavelengths were removed. However, the subsequent increasing in RMSECV values could be attributed to the elimination of key wavelengths. The optimal subset of wavelengths, marked with an asterisk, was chosen based on the minimum 5‐fold RMSECV value. In Figure 5c, each line represents the coefficient recorded for each wavelength in the different sampling runs. A larger absolute coefficient indicated a higher probability of selecting the corresponding wavelength. Therefore, a subset of wavelengths, along with their regression coefficients, could be picked up from each sampling run. The optimal subset, which corresponding to the minimum 5‐fold RMSECV value, was marked with an asterisk on the vertical line. As calculated by CARS, the RMSECV achieved its minimum value at 27 sampling runs, corresponding to a total of 37 wavelengths (accounting for 3.61% of the full spectra). The feature wavelengths chosen for predicting the MC by CARS were presented in Table 2. And the performance of PLSR, MLR, ELM, and BP models, developed using the selected effective wavelengths, was presented in Table 3.

**TABLE 2:** Optimal wavelengths for predicting the MC selected by CARS and RF.

Methods	Band no.	Wavelength (nm)
CARS	37	209.61, 211.24, 228.38, 234.09, 242.23, 243.86, 245.49, 249.55, 252.80, 253.62, 256.86, 272.28, 273.90, 277.14, 299.78, 310.27, 324.77, 334.42, 337.63, 350.47, 357.69, 358.49, 376.89, 380.08, 387.26, 468.19, 468.98, 472.93, 473.72, 897.57, 898.32, 911.73, 913.21, 957.74, 960.70, 961.43, 968.83
RF	16	473.72, 610.44, 809.09, 834.69, 866.19, 868.44, 876.67, 897.57, 931.80, 943.66, 946.63. 954.04, 961.43, 979.90, 987.28, 997.59

**TABLE 3:** Prediction results of the PLSR, MLR, ELM, and BP models.

Models	Methods	Band no.	Calibration set		Prediction set		
			*R* ^2^_C_	RMSEC	*R* ^2^_P_	RMSEP	RPD
PLSR	CARS	37	0.90	0.47	0.84	0.46	2.51
	RF	16	0.68	0.85	0.66	0.65	1.75
MLR	CARS	37	0.91	0.45	0.81	0.49	2.33
	RF	16	0.68	0.85	0.66	0.65	1.75
ELM	CARS	37	0.80	0.67	0.75	0.56	2.04
	RF	16	0.73	0.78	0.69	0.62	1.84
BP	CARS	37	0.82	0.63	0.67	0.64	1.78
	RF	16	0.66	0.84	0.63	0.69	1.66

As shown in Table 3, comparing the performances of the PLSR, MLR, ELM, and BP neural network models established based on feature variables by CARS, the CARS‐PLSR model exhibited better results for predicting the MC, with *R* ^2^_P_, RMSEP, and RPD values of 0.84, 0.46, and 2.51, respectively.

### 3.4 Modeling based on feature variables by RF

**FIGURE 6:**
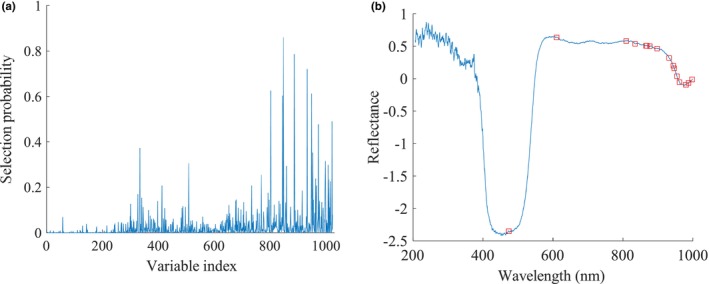
Processing of RF for the MC. (a) The probability of selecting each wavelength, (b) The selected variables.

For RF, a higher selection probability indicated a greater significance of the corresponding wavelengths. In this study, the RF algorithm was set to run 1000 operations, the number of potential variables was 14, and the initial number of sampled variables was 2. The probability of selecting each wavelength by RF for assessing the MC of loquats was shown in Figure 6a. The selected variables, marked as hollow red squares, were shown in Figure 6b.

As shown in Figure 6a, only a small fraction of the wavelengths demonstrated high selection probabilities, while the majority exhibited low selection probabilities, which indicated that there was a lot of irrelevant information in the full spectrum data. In this study, a total of 16 feature wavelengths were selected (accounting for 1.56% of the full spectrum). Table 2 presented the specific wavelengths chosen for predicting the MC by RF.

**FIGURE 7:**
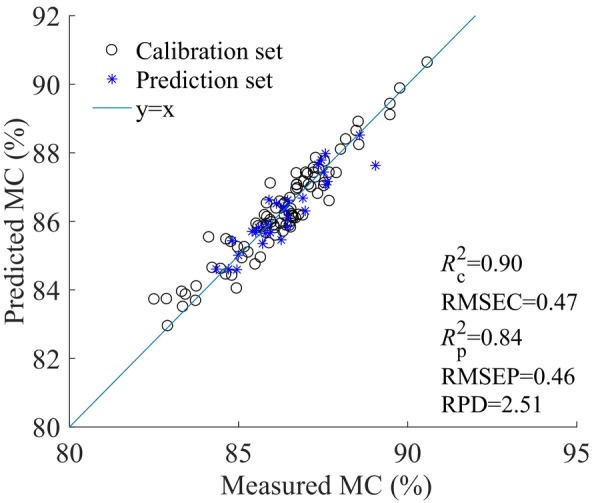
Scatter plots of the modeling results of the CARS‐PLSR model.

Furthermore, Table 3 showed the performance of PLSR, MLR, ELM, and BP models developed by using the selected effective wavelengths. As shown in Table 3, the RF‐ELM model outperformed the PLSR, MLR, and BP neural network models constructed using feature variables by RF, with corresponding values of 0.69, 0.62, and 1.84 for *R* ^2^_P_, RMSEP, and RPD, respectively. And then comparing the models established based on the variable selected by CARS and RF, the performance of the prediction models built on CARS outperformed those constructed using RF. Particularly, CARS‐PLSR model for predicting MC exhibited the best prediction performance, with *R* ^2^_P_ and RPD values of 0.84 and 2.51, respectively. The RPD value exceeding 2.5 indicated the excellent performance of the CARS‐PLSR model. Figure 7 presented the scatter plots between the measured and predicted values. The formulae presenting the optimal CARS‐PLSR detection model for the MC of loquats was as follows (Equation 9).

(9) $$\begin{array}{cc} Y_{\mathrm{MC}}= & 86.27-0.31\lambda_{209.61}+0.39\lambda_{211.24}-0.45\lambda_{228.38}+0.21\lambda_{234.09}-0.33\lambda_{242.23}\\ & -0.32\lambda_{243.86}+0.17\lambda_{245.49}-0.43\lambda_{249.55}+0.18\lambda_{252.80}+0.51\lambda_{253.62}\\ & +0.35\lambda_{256.86}+0.39\lambda_{272.28}-0.40\lambda_{273.90}-0.45\lambda_{277.14}+0.64\lambda_{299.78}\\ & -0.64\lambda_{310.27}+0.37\lambda_{324.77}+0.49\lambda_{334.42}-0.56\lambda_{337.63}+0.48\lambda_{350.47}\\ & -0.36\lambda_{357.69}-0.22\lambda_{358.49}+0.46\lambda_{376.89}+1.40\lambda_{380.08}-2.39\lambda_{387.26}\\ & +0.26\lambda_{468.19}-0.62\lambda_{468.98}-0.27\lambda_{472.93}-0.15\lambda_{473.72}+0.64\lambda_{897.57}\\ & +0.34\lambda_{898.32}+0.12\lambda_{911.73}+0.06\lambda_{913.21}-0.67\lambda_{957.74}-0.68\lambda_{960.70}\\ & -0.52\lambda_{961.43}-0.14\lambda_{968.83} \end{array}$$

where, *Y*_MC_ is the predicted values for the MC. *λ*_*i*_ is the reflectance at the characteristic wavelength, with the subscript *i* denoting the wavelength (nm).

### 3.5 Freshness level classification

**FIGURE 8:**
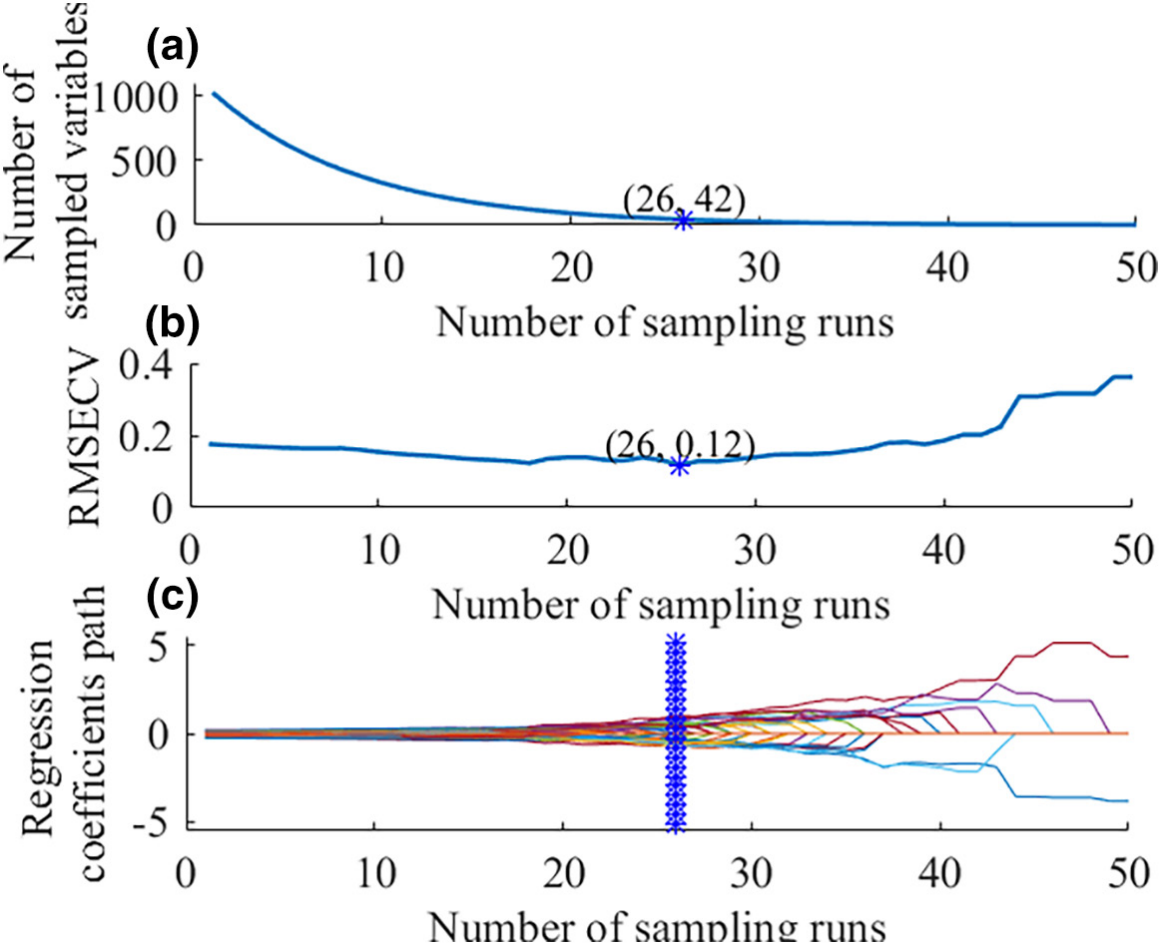
Processing of CARS for the classification. The change trend of the variables number (a), 5‐fold RMSECV values (b), and the regression coefficient path (c) with the increase of Monte‐Carlo sampling runs.

**FIGURE 9:**
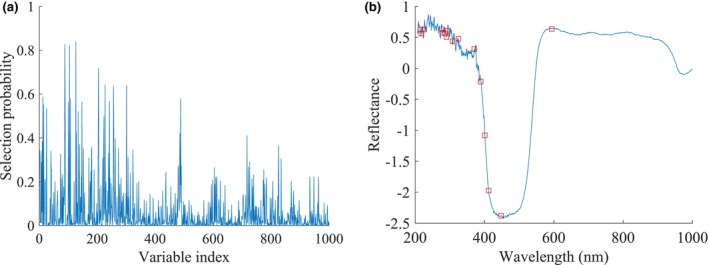
Processing of RF for the classification. (a) The probability of selecting each wavelength, (b) The selected variables.

Figures 8 and 9 showed the variable selection processing for CARS and RF, respectively. The optimal wavelengths for freshness classification selected by CARS and RF are given in Table 4. As shown in Figure 8, the RMSECV achieved minimum value at 26 sampling runs, corresponding to a total of 42 wavelengths (4.10% of the full spectra). As shown in Figure 9, a total of 16 effective wavelengths were selected (1.56% of the full spectra) using RF. Table 4 presented the specific wavelengths chosen for freshness classification selected by CARS and RF.

**TABLE 4:** Optimal wavelengths for freshness classification selected by CARS and RF.

Methods	Band no.	Wavelength (nm)
CARS	42	212.06, 216.96, 221.86, 238.98, 239.79, 241.42, 246.30, 247.93, 248.74, 254.43, 256.86, 263.36, 267.42, 276.33, 283.62, 285.23, 286.04, 286.85, 290.09, 292.51, 293.32, 295.74, 307.85, 317.52, 324.77, 329.60, 347.26, 352.08, 356.88, 369.69, 370.49, 371.29, 377.68, 381.68, 383.27, 385.67, 400.81, 405.59, 406.39, 411.95, 412.75, 414.34
RF	16	215.33, 217.78, 225.94, 277.14, 277.95, 286.85, 290.09, 292.51, 307.85, 324.77, 370.49, 388.86, 400.81, 411.95, 447.65, 594.16

The MC is an essential quality factor of fruits, the higher the MC, the fresher the fruit (Kammar et al., 2016). As shown in Section (Statistics of moisture content), the mean value of the MC decreased with prolonged storage time, indicating a gradual decline in the freshness of the loquat fruit. In this study, the freshness of loquats was defined as four levels (Level I, Level II, Level III, and Level IV) according to storage time. A total of 120 loquats were divided into the calibration set (*n* = 90) and the prediction set (*n* = 30) with a 3:1 ratio using the KS algorithm. Freshness recognition models, including SKNN, SVM, and PLS‐DA, were established using characteristic variables selected by CARS and RF. The classification results were summarized in Table 5.

**TABLE 5:** Classification results of freshness of loquats by SKNN, SVM, and PLS‐DA models.

Models	Methods	Band no.	Calibration set			Prediction set			Total accuracy (%)
			Number	Error	*I*_R_ (%)	Number	Error	*I*_R_ (%)	
SKNN	CARS	42	90	0	100	30	1	96.67	99.17
	RF	16	90	5	94.44	30	2	93.33	94.17
SVM	CARS	42	90	0	100	30	0	100	100
	RF	16	90	4	95.56	30	0	100	96.67
PLS‐DA	CARS	42	90	0	100	30	1	96.67	99.17
	RF	16	90	8	91.11	30	1	96.67	92.50

**FIGURE 10:**
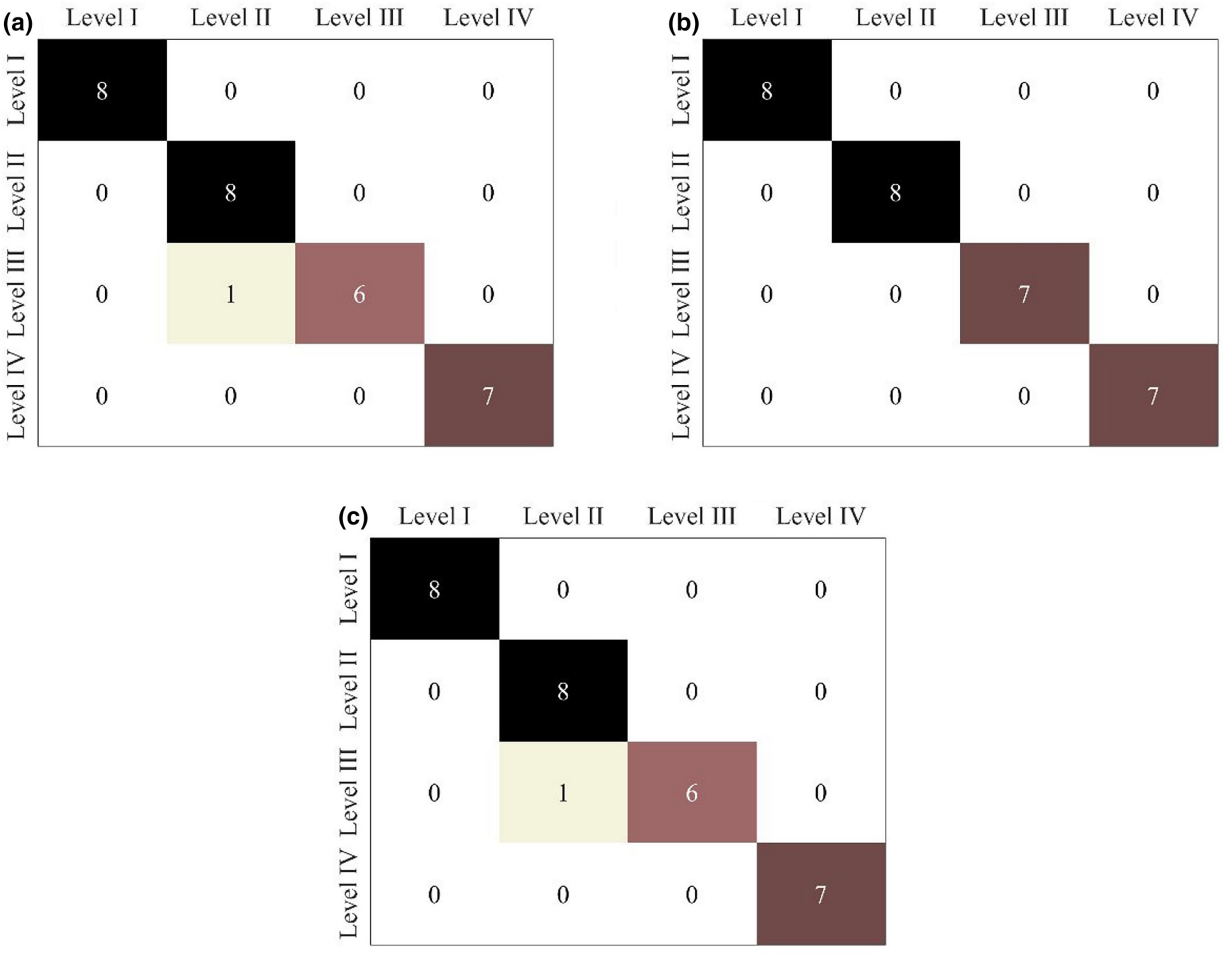
Confusion matrix in the prediction set for SKNN (a), SVM (b), and PLS‐DA models (c).

As shown in Table 5, discrimination models built on CARS exhibited higher accuracy compared to those constructed using RF. Especially, the CARS‐SVM model had a highest discrimination accuracy for the prediction set. As illustrated in Figure 10, both the CARS‐SKNN and CARS‐PLS‐DA models misclassified one Level III sample in the prediction set as Level II, resulting in one misclassifications. In contrast, the SVM model exhibited no classification errors. In summary, the CARS‐SVM model demonstrated superior performance compared to the other models in determining the freshness of loquats, with a discrimination accuracy of 100% for both the calibration and prediction sets.

## 4 CONCLUSIONS

A novel strategy based on optical fiber spectroscopy in the range of 200–1000 nm was employed to determine the MC and the freshness level in loquats. CARS and RF was used to identify the optimal band combination capable of reflecting changes in sample characteristic information within spectral curves affected by overlapping and substantial noise. On this basis, prediction models for the MC were developed using PLSR, MLR, ELM, and BP neural network algorithms, while freshness level discrimination models were established using SKNN, SVM, and PLS‐DA methods. The results showed that the model built using characteristic variables selected by CARS exhibits superior performance in comparison to the model built using characteristic variables selected by RF. And the CARS‐PLSR model achieved satisfactory prediction results for predicting the MC using only approximately 3.61% variables of the full spectrum, with *R* ^2^_P_ of 0.84 and RPD of 2.51. Regarding the discrimination models, the CARS‐SVM model obtained relatively best discrimination performance, which achieving 100% accuracy for both the calibration and prediction sets. The study indicates that the use of optical fiber spectroscopy technology combined with chemometrics for rapid and non‐destructive determination of the MC and freshness for loquats is feasible.

## AUTHOR CONTRIBUTIONS

**Qinglong Meng:** Conceptualization (equal); funding acquisition (equal); project administration (equal); validation (equal); writing – original draft (equal); writing – review and editing (equal). **Shunan Feng:** Conceptualization (equal); data curation (equal); investigation (equal); methodology (equal); writing – original draft (equal); writing – review and editing (equal). **Tao Tan:** Data curation (equal); formal analysis (equal); methodology (equal); software (equal). **Qingchun Wen:** Data curation (equal); formal analysis (equal); investigation (equal). **Jing Shang:** Funding acquisition (equal); project administration (equal); resources (equal); supervision (equal); validation (equal); writing – review and editing (equal).

## CONFLICT OF INTEREST STATEMENT

All the authors declare that they have no conflict of interest.

## ETHICS APPROVAL STATEMENT

This study does not involve any human or animal testing.

## PRACTICAL APPLICATIONS

The MC and freshness are critical factors in determining the quality of loquats. Conventional methods for assessing the MC and freshness are typically destructive and time‐consuming. Due to the remarkably short shelf‐life of loquats, it is crucial to rapidly detect their MC and freshness for evaluating their commercial value and shelf‐life. Optical fiber spectroscopy technology presents itself as an attractive non‐destructive technique for assessing the quality of food products. The presented findings indicate that optical fiber spectroscopy has significant potential for online monitoring of the MC and freshness of loquats.

## Data Availability

The data that support the findings of this study are available from the corresponding author upon reasonable request.
